# Use of a universal targeting CAR T cell to simultaneously kill cancer cells and cancer-associated fibroblasts

**DOI:** 10.3389/fimmu.2025.1539265

**Published:** 2025-02-17

**Authors:** Bo Huang, Suilan Zheng, Kasireddy Sudarshan, Ramesh Mukkamala, Madduri Srinivasarao, Tushar Sardesai, Xiaofei Yang, Haiyan Chu, Philip S. Low

**Affiliations:** ^1^ Department of Chemistry and Institute for Drug Discovery, Purdue University, West Lafayette, IN, United States; ^2^ Research & Translational Sciences, Umoja Biopharma, Seattle, WA, United States

**Keywords:** universal CAR T cells, tumor microenvironment, cancer-associated fibroblasts (CAFs), solid tumors, fibroblast activation protein (FAP)

## Abstract

CAR T cells therapies have demonstrated success in treating hematologic malignancies, but have proven less effective in eradicating solid tumors. While suppressive immune cells may contribute to reduced CAR T cell efficacies in malignant masses, cancer-associated fibroblasts (CAFs) are also believed to facilitate tumor survival by secreting growth factors, immunosuppressive cytokines, and extracellular matrix components that inhibit drug and immune cell filtration and facilitate metastasis. In an effort to eliminate both CAFs and cancer cells simultaneously, we have employed a universal CAR T cell that can attack both cell types when supplemented with appropriate bispecific adapters. We show here that tumor regression is indeed enhanced when CAR T cells are directed to concurrently kill both cancer cells and CAFs. We further demonstrate that simultaneous targeting of both cell types enhances CAR T cell proliferation, activation, tumor infiltration, and tumor distribution relative to targeting only a single cell type. Because all of these benefits are achieved in both cold and hot tumors without significant toxicity, we conclude that use of a universal CAR T cell in combination with multiple bispecific adapters can provide a safe, potent, cost-effective, and scalable alternative to the treatment of solid tumors with conventional CAR T cells.

## Introduction

Although chimeric antigen receptor (CAR) T cell therapies have proven effective in treating liquid tumors (1–4), their potencies in eradicating solid tumors have been less impressive (5–8). Reasons for this reduced potency have included poor CAR T cell penetration into tumor masses (5, 9), exhaustion of CAR T cells induced by chronic antigen exposure (10–12), selection for cancer cells that lack the targeted antigen (13–15), and/or an immunosuppressive tumor microenvironment generated by tumor stromal cells (16–18). Cancer-associated fibroblasts (CAFs) are believed to contribute to this CAR T cell inactivation by releasing immunosuppressive cytokines such as IL-10 and TGF-β (19), secreting tumor-stimulating growth factors including fibroblast growth factors, vascular endothelial growth factors, and hepatocyte growth factor (19–22), and depositing extracellular matrix components such as collagen and fibronectin that can create an impermeable barrier to entry of immune cells and therapeutic agents, and also facilitate cancer metastasis (19, 21, 23). Not surprisingly, CAF contents in solid tumors correlate inversely with overall survival (19, 24, 25).

Strategies to reduce the number of CAFs in solid tumors have exploited the upregulation of fibroblast activation protein (FAP), a cell surface serine protease, primarily on CAFs in solid tumors (26, 27). In these approaches, the over-expressed FAP has been targeted with cytotoxic drugs (28–31), radioligand therapies (27, 32–35), CAR T cell therapies (36–43), and/or bispecific T cell engagers (44–46). While each of these therapies may enhance tumor regression, many have suffered from insufficient potency or dose-limiting toxicity that has prevented their adoption in the clinic (42, 43). Although infusion of a second CAR T cell directed against CAFs has recently yielded encouraging results (36–38), this approach has required the engineering and expansion of a second CAR T cell preparation from the same patient that may render widespread adoption more difficult (47–49).

**Figure 1:**
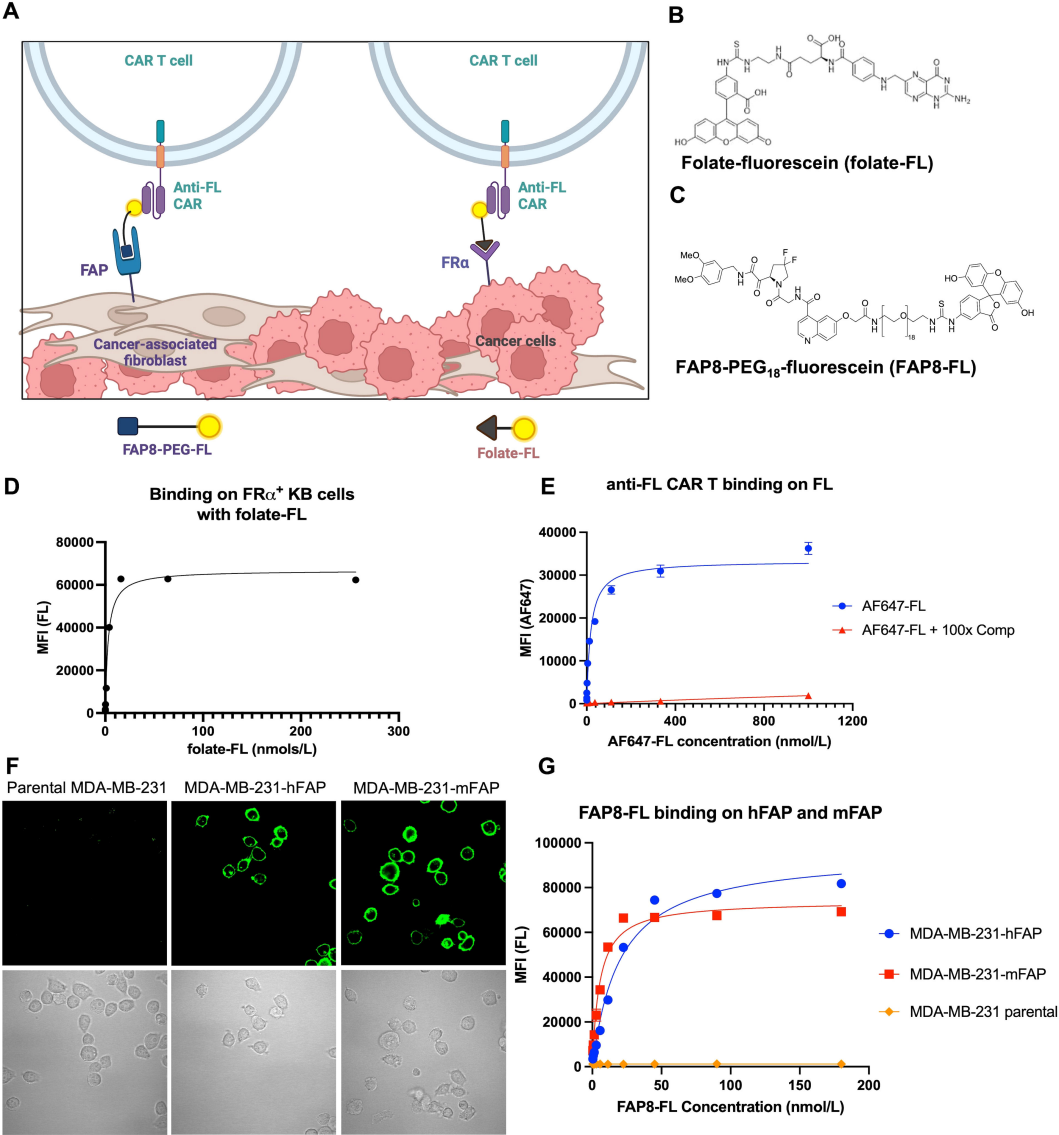
Development of a FAP8-FL bispecific adapter. **(A)** Sketch depicting the use of the same universal CAR T cell to engage both cancer cells and CAFs in a solid tumor. Created with BioRender.com. **(B)** Structure of folate-FL. **(C)** Structure of FAP8-FL. **(D)** Binding affinity of folate-FL for FRα-expressing KB cancer cells. **(E)** Binding affinity of AlexaFluor647-fluorescein conjugate to anti-FL CAR T cells in the absence (AF647-FL) or presence (AF647-FL + comp) of 100-fold molar excess of fluorescein. **(F)** Binding of FAP8-FL to unmodified MDA-MB-231 cells (left panel) or MDA-MB-231 cells transduced with human FAP (middle panel) or murine FAP (right panel), and **(G)** binding analysis of FAP8-FL to these same cells determined by flow cytometry. Mean ± SD, n=3.

As a remedy to this problem, we have explored the use of a universal CAR T cell in which the CAR is comprised of a single chain variable fragment (scFv) that binds fluorescein rather than a tumor antigen (50–52). In this approach, the anti-fluorescein CAR T cell is induced to engage the cancer cell by injection of a bispecific adapter comprised of fluorescein linked to a low molecular weight tumor-targeting ligand that can bridge between the CAR T cell and cancer cell (**Figure 1A**). Formation of this bridge triggers killing of the cancer cell by the CAR T cell along with the subsequent proliferation of the CAR T cell. While the flexibility of this approach has enabled the use of two different bispecific adapters to force engagement of a universal CAR T cell with two antigenically orthogonal cancer cells in the same tumor mass (52), the approach has never been explored for its ability to enable simultaneously killing of both cancer cells and stromal cells. In the study below, we explore the ability of this universal CAR T cell to concurrently eliminate cancer cells and CAFs from the same tumor mass.

For engagement of the cancer cells, we exploit folate receptor alpha (FRα) that is over-expressed on ~40% of human cancers (53) and induce cancer cell engagement with the CAR T cells by injection of a folate-fluorescein (folate-FL) bispecific adapter. For killing of the CAFs we employ an analogous FAP8-fluorescein (FAP8-FL) bispecific adapter that similarly bridges between CAFs and the CAR T cells. Importantly, concurrent administration of both bispecific adapters is found to significantly enhance tumor regression relative to injection of either adapter alone, i.e. demonstrating that the universal CAR T cell has the ability to kill both cells types concurrently and that elimination of CAFs enhances CAR T cell killing of cancer cells.

## Results

### Design and evaluation of bispecific adapters binding

In order to evaluate the ability of a single anti-fluorescein (anti-FL) CAR T cell to kill both cancer cells and cancer-associated fibroblasts (CAFs) in the same tumor mass, we required two different bispecific adapters that would mediate engagement of our universal CAR T cell with each cell type. For CAR T cell killing of the folate receptor (FRα) over-expressing cancer cells, we employed the folate-fluorescein (folate-FL) bispecific adapter shown in (**Figure 1B**; **Supplementary Figure S1**), (50, 51, 54). Binding of this adapter to both FRα on the cancer cells and the anti-FL CAR on the CAR T cells was found to occur with high affinity, i.e. dissociation constants of ~3 nM and ~20 nM, respectively (**Figures 1D, E**).

A similar bispecific adapter to tether the same anti-FL CAR T cells to CAFs was then synthesized by attaching a fibroblast activation protein (FAP) ligand [FAP8 (55)] to fluorescein via a 54 atom PEG_18_ spacer (**Figure 1C**). This extended spacer was selected because shorter spacers were unable to mediate bridging of the CAR T cell to a FAP-expressing cell, and significantly longer spacers were found to display reduced affinities. Binding of FAP8-fluorescein (FAP8-FL) to cells transduced to express either human or murine FAP displayed strong cell surface localization (**Figure 1F**) characterized by dissociation constants of 20 nM for human FAP and 5 nM for murine FAP (**Figure 1G**). Since no binding to cells lacking FAP could be detected, we concluded that binding of this bispecific adapter was FAP specific.

### FAP8-FL mediates potent and specific killing of FAP-expressing cells

**Figure 2:**
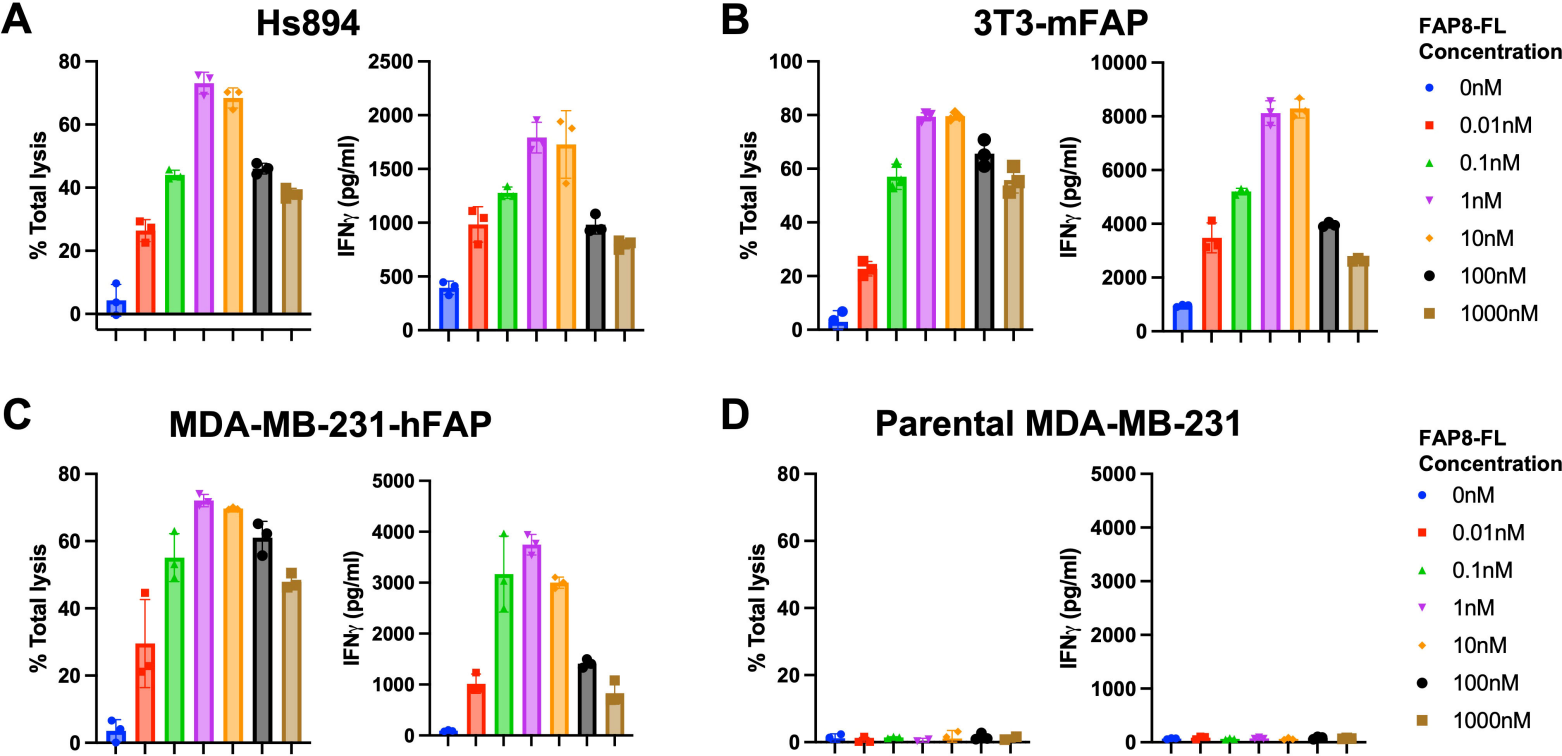
Evaluation of FAP8-FL mediated anti-FL CAR T cell engagement of different FAP-expressing cell lines. Percent cell lysis (left) and IFNγ release (right) were measured in a coculture killing assay in which the target cells were **(A)** Hs894 human CAFs, **(B)** 3T3 murine fibroblasts transduced with mouse FAP, **(C)** MDA-MB-231 cells transduced with human FAP, or **(D)** parental MDA-MB-231 with no FAP. Mean ± SD, n=3.

To determine whether FAP8-FL might mediate CAR T cell killing of FAP-expressing cells *in vitro*, we incubated several FAP-expressing cell lines with both our universal CAR T cell and our FAP-linked bispecific adapter (**Supplementary Figures S2**, **S3**), with the relative FAP expression of these cell lines determined by anti-FAP antibodies (**Supplementary Figure S4**). As shown in **Figure 2**, FAP8-FL readily induced anti-FL CAR T cell killing of a human fibroblast cell line (**Figure 2A**), a mouse fibroblast cell line (**Figure 2B**), and MDA-MB-231 cells transduced to express human FAP (**Figure 2C**). Moreover, as FAP8-FL concentration was increased, cell killing first increased and then decreased in the predictable bell-shaped manner (i.e. due to eventual saturation of both fluorescein and FAP binding sites with different bispecific adapters). Since maximum killing potency was observed at ~1 nM adapter and since killing was always accompanied by release of IFNγ, we conclude that FAP8-FL mediates CAR T cell activation and the consequent killing of FAP-expressing cells with high potency. Because FAP-negative (nontransduced) MDA-MB-231 cells were not killed upon addition of FAP8-FL (**Figure 2D**), the data further demonstrate that killing was FAP-specific.

### Simultaneous targeting of cancer cells and CAFs enhances tumor suppression

**Figure 3:**
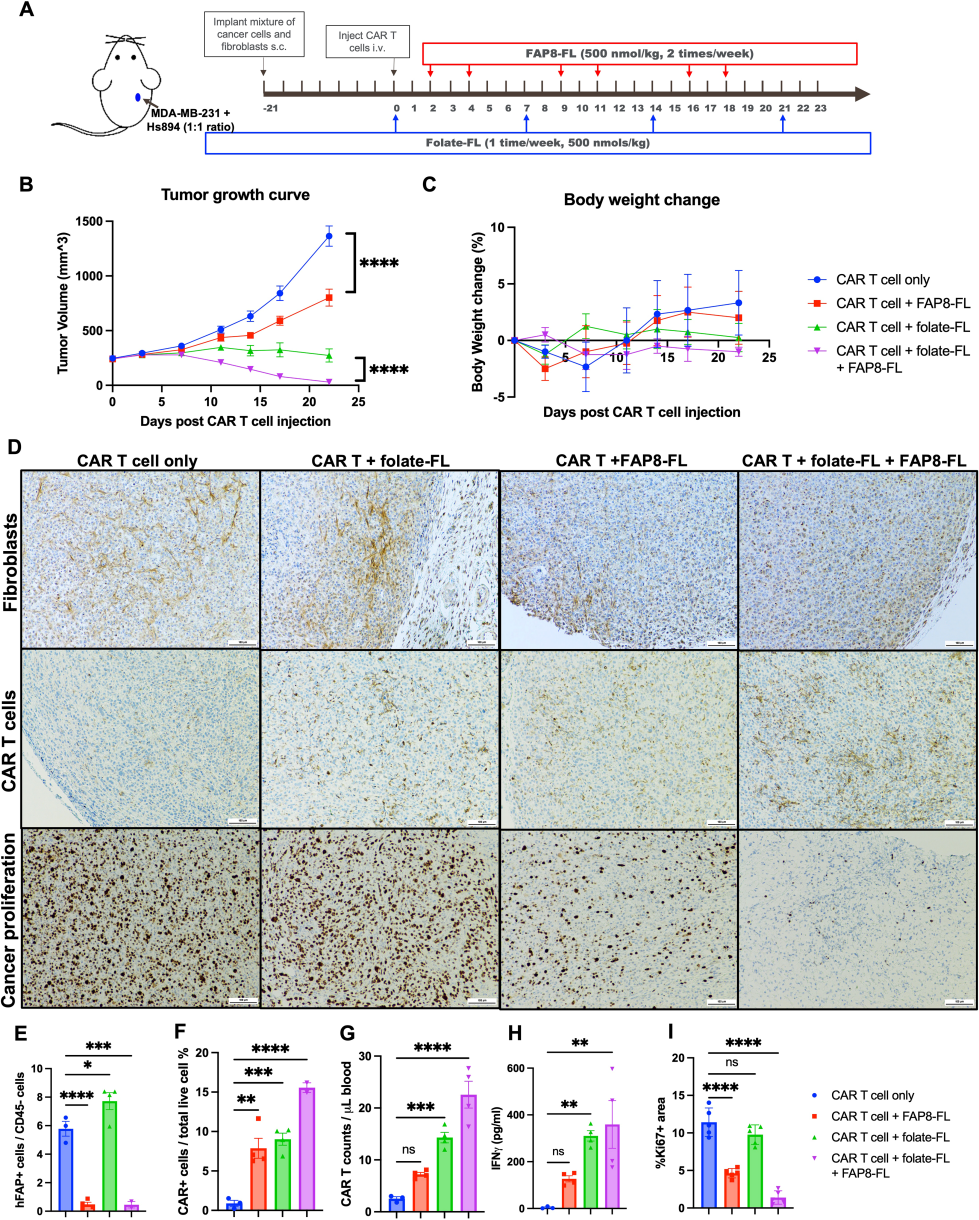
Evaluation of the ability of the same anti-FL CAR T cells to eradicate both cancer cells and CAFs in the same tumor mass and its impact on solid tumor. **(A)** Tumors were created by subcutaneous (s.c.) injection of a mixture of parental FRα+ MDA-MB-231 cells and Hs894 fibroblasts and allowed to grow to ~250 mm^3^ prior to injection of 3 million CAR T cells per mouse and dosing with bispecific adapters, as indicated. **(B)** Evaluation of tumor growth versus time in the different treatment groups. **(C)** Mouse body weights in the same study. **(D)** IHC staining of representative tumor sections obtained from the same treatment groups on day 23 for fibroblasts (murine FAP), human CAR T cells (human CD3), and cancer proliferation (Ki67). Tumors and peripheral blood samples collected from mice were quantitated for **(E)** residual Hs894 human FAP-expressing fibroblasts in the tumors (flow cytometry of digested tumors), **(F)** human CAR T cells in the same tumors (flow cytometry of digested tumors), **(G)** CAR T cells in the blood (flow cytometry), **(H)** IFNγ as a measure of CAR T cell activation (ELISA quantitation in mouse blood), and **(I)** Ki67 in the same tumors [ImageJ quantitation of stained sections (60) in panel **(D)**]. Mean ± SEM, n=3 in CAR T cell only group and n=4 in all treatment groups. Statistical significance between groups was determined using two-way ANOVA in **(B)** (****P < 0.0001) and using one-way ANOVA (*P < 0.05, **P<0.01, ***P < 0.001, ****P < 0.0001) in **(E-I)**.

To evaluate whether the anti-FL CAR T cells might be capable of simultaneously eradicating both cancer cells and CAFs in the same tumor mass, we co-injected NSG mice with both unmodified (parental MDA-MB-231) cells that express no FAP and human Hs894 fibroblasts that naturally express FAP (**Figure 3A**) based on similar approach by others to build a physiologically relevant CAFs-containing human tumor in mice (36). After allowing the tumors to reach ~250 mm^3^, mice were injected intravenously with either CAR T cells alone or CAR T cells in combination FAP8-FL, folate-FL, or both adapters together. Folate-FL was dosed at once per week based on previously optimized dosing frequency (50), and FAP8-FL was dosed with at least one day gap to allow excess adapter excrete out of mouse bloodstream. As seen in **Figure 3B**, administration of FAP8-FL alone induced a small but statistically significant decrease in tumor growth, suggesting that elimination of fibroblasts may suppress tumor growth by killing the fibroblasts. Injection of folate-FL, in contrast, promoted a more profound reduction in tumor size, arguing that direct CAR T cell attack on the FRα-expressing MDA-MB-231 cells more effectively inhibited tumor growth. Importantly, concurrent treatment with both bispecific adapters induced nearly complete tumor eradication, demonstrating that diversion of some universal CAR T cells to engage FAP-expressing stromal cells (i.e. CAFs) not only did not hinder but actually augmented their abilities to inhibit tumor growth. Moreover, because tumor suppression could be achieved with no weight loss (**Figure 3C**), the data suggest that any FAP-expressing fibroblasts that might have been killed in healthy tissues must have occurred with little overt toxicity.

To obtain a more mechanistic understanding of the processes that might have contributed to the improved efficacy in the presence of both adapters, we analyzed the changes in CAR T cell and fibroblast abundances in all treatment groups by both immunohistochemistry of tumor sections and flow cytometry of isolated tumor cells (see Methods). As shown in **Figure 3D**, fibroblasts remained abundant in tumors from mice treated with either CAR T cells alone or CAR T cells plus folate-FL, but were largely eliminated from tumors in both cohorts treated with the FAP8-FL adapter. These data thus confirm that treatment with CAR T cells + FAP8-FL kills CAFs in solid tumors, and this conclusion is further supported by the flow cytometry data in **Figure 3E**; **Supplementary Figure S5**.

Comparison of CAR T cell abundances in the different treatment groups then revealed that administration of CAR T cells plus FAP8-FL increased CAR T cell proliferation and infiltration relative to cohorts not treated with the FAP8-FL adapter. Thus, CAR T cell numbers in the tumors (**Figure 3F**; **Supplementary Figure S6**) and peripheral blood (**Figure 3G**; **Supplementary Figure S7**) were both increased in mice treated with CAR T cells + FAP8-FL relative to CAR T cells alone. CAR T cells were also elevated in mice treated with CAR T cells + FAP8-FL + folate-FL relative to mice treated with CAR T cells + folate-FL alone. Taken together these data establish that co-administration of FAP8-FL adapter with folate-FL adapter significantly augments infiltration of the CAR T cells into tumor masses. The data also argue that any competition between the FAP8-FL and folate-FL for binding to the anti-FL CAR must be minimal, since tumor eradication (**Figure 3A**), CAR T cell infiltration (**Figure 3F**), and IFNγ release (**Figure 3H**) all improved by concurrent administration of the two adapters. In fact, the data on IFNγ release may be an underestimate of the true impact of dual adapter administration, since the tumors had already been eradicated in the two mice with low IFNγ levels, suggesting that their IFNγ levels may have already been returning to normal when their peripheral blood samples were collected.

To assess the impact of CAF elimination on cancer cell proliferation, we next quantitated Ki67 staining of multiple tumor sections from the same treatment groups using ImageJ analysis. As shown in **Figure 3I**, injection of FAP8-FL adapter invariably reduced cancer cell proliferation relative to similar treatment groups lacking FAP8-FL. Although already reported by others (38, 40), these data confirm that CAFs can promote cancer cell proliferation, presumably by their release of growth factors (19–22), and that elimination of CAFs thereby suppresses tumor growth.

### Dual targeting overcomes CAF-barriers in immunologically cold tumors

**Figure 4:**
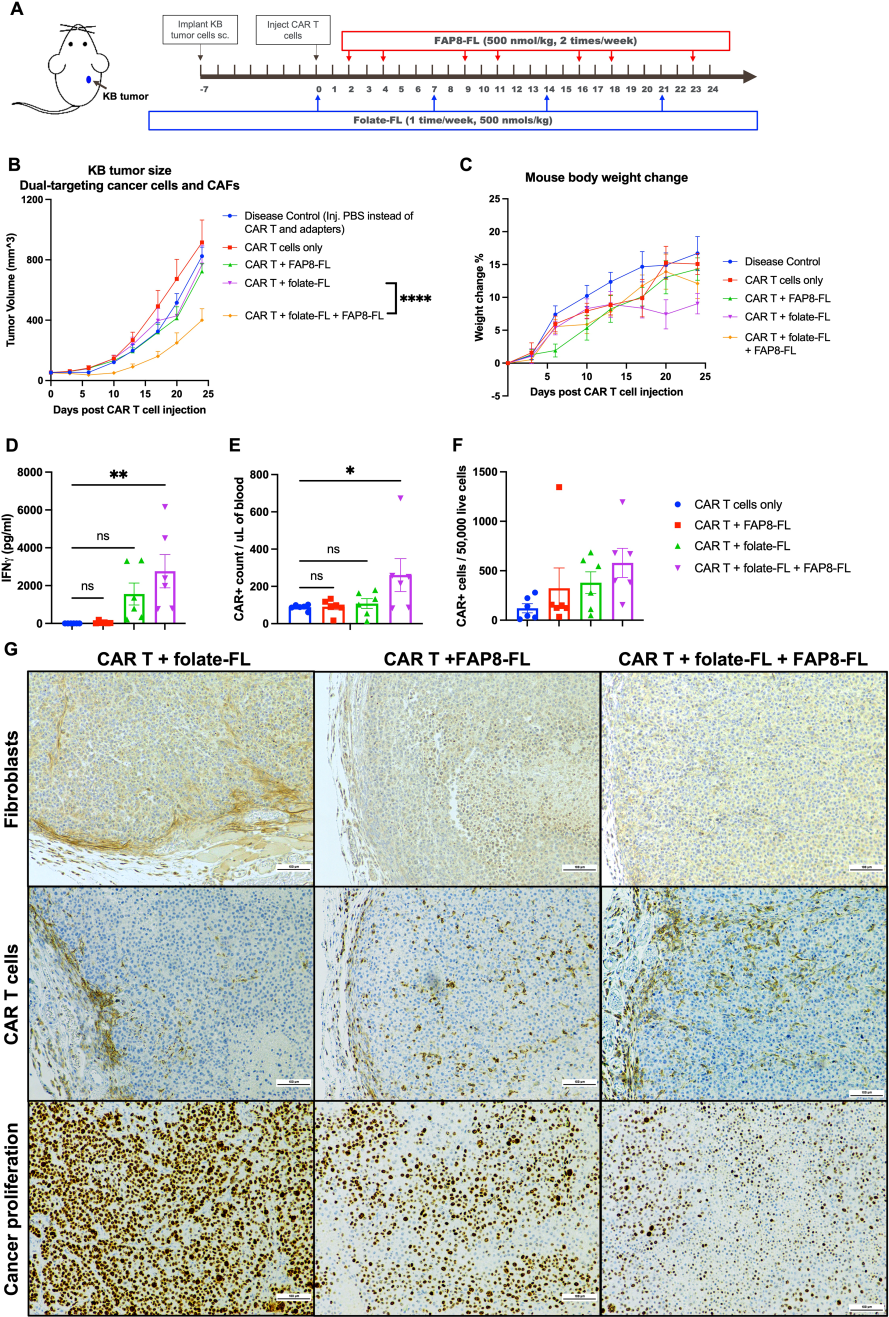
Evaluation of the abilities of the same anti-FL CAR T cells to target both cancer cells and CAFs in the same immunologically cold KB tumors. **(A)** KB tumors were implanted by s.c. injection of cultured KB cells into NSG mice and allowed to grow to ~50 mm^3^ prior to injection of 8 million CAR T cells per mouse and dosing with bispecific adapters, as indicated. **(B)** Evaluation of tumor growth versus time. **(C)** Evaluation of body weight changes versus time. **(D)** Blood CAR T cell counts were quantitated by flow cytometry, and **(E)** peripheral blood IFNγ concentrations were determined by ELISA at the study endpoint as a measure of T cell activation. **(F)** CAR T cell infiltration into the tumors was assessed by flow cytometry of dissociated tumors. **(G)** Distributions of fibroblasts (stained with anti-mouse FAP antibody), CAR T cells (anti-human CD3 antibody), and proliferating cancer cells (Ki67 antibody) in tumor sections were determined by IHC staining. Mean ± SEM, n=6, statistical significance between groups **(B)** was determined using two-way ANOVA (****P < 0.0001), and between groups **(D, E)** was determined using one-way ANOVA (*P < 0.05, **P<0.01).

Next, because both endogenous immune cells and exogenous CAR T cells are frequently excluded from immunologically “cold” tumors, at least in part due to the “barriers” created by accumulation of CAFs near the tumor periphery (19, 38, 40), the question arose whether a universal CAR T cell in combination with both a CAF- and cancer cell-killing bispecific adapter might more effectively penetrate and destroy these resistant tumors than a CAR T cell capable of killing only one tumor cell type. Although MDA-MB-231 tumors have been shown to be readily eradicated by our universal CAR T cells in combination with folate-FL adapters, similarly treated KB tumors have been found to be extremely resistant (12). To obtain an initial indication of whether this difference in CAR T cell responsiveness might have derived from a difference in CAF content/distribution, we first stained both KB and MDA-MB-231 tumors for CAR T cells and CAFs to evaluate their distributions and abundances when treated with CAR T cells plus folate-FL adapter. As shown in **Supplementary Figure 8**, CAR T cells are highly enriched and uniformly distributed in the MDA-MB-231 tumors, but few in number and concentrated at the periphery in KB tumors. Moreover, consistent with previous observations of immunologically cold tumors (12, 38, 40), CAFs are more abundant and peripherally confined in KB tumors (**Supplementary Figure S8**), but less numerous and more evenly distributed in MDA-MB-231 tumors. Based on suggestions by others that this barrier-like distribution of fibroblasts restricting CAR T cell penetration defines an immunologically cold tumor (19, 21, 38), we elected to test the ability of our universal CAR T cells to concurrently eradicate both cancer cells and CAFs in these cold KB tumors (**Figure 4A**). As shown in **Figure 4B**, neither the CAR T cells plus folate-FL (targeting the cancer cells) nor CAR T cells plus FAP8-FL (targeting the CAFs) alone exerted an impact on KB tumor growth, i.e. consistent with their immunologically cold nature. However, the combination of CAR T cells with both bispecific adapters promoted an ~50% suppression of tumor growth. The fact that co-administration of both adapters also induced the largest IFNγ release (**Figure 4D**) and proliferation of CAR T cells (**Figures 4E, F**) further emphasized the benefit that the two bispecific adapters provided in treating the cold KB tumors. Because body weights throughout the study (**Figure 4C**) increased, regardless of the adapter combination used, we surmise that all combinations analyzed were also largely nontoxic.

To determine whether the peripherally confined distribution of CAR T cells and CAFs characteristic of cold KB tumors may have been at least partially disrupted by the above therapy, we next stained sections from all of the tumors for CAFs and CAR T cells (**Figure 4G**). As anticipated, CAR T cells were largely limited to tumor boundaries in the untreated specimens, but encouragingly distributed throughout the tumors in mice treated with either FAP8-FL alone or FAP8-FL + folate-FL. Since CAFs were largely absent in both of these latter treatment cohorts, it could be concluded that elimination of CAFs can permit greater penetration of CAR T cells into solid KB tumors, i.e. perhaps by removing the physical barriers created by the CAFs. However, the fact that CAR T cell infiltration is even more improved by simultaneous targeting of both tumor cells and CAFs further suggests that the KB cells may also contribute to the confinement of CAR T cells to the tumor periphery.

Finally, since CAFs secrete growth factors that promote tumor growth (19–22), we decided to assess the impact of FAP8-FL-directed CAR T cell treatment on cancer cell proliferation. As shown in the lower panel of **Figure 4G**, a remarkable reduction in the cell proliferation marker (Ki67) was seen upon targeting of the CAFs alone and this was further suppressed when both bispecific adapters were administered, i.e. confirming that CAFs indeed contribute to cancer cell proliferation and that dual targeting of both CAFs and cancer cells is more effective than targeting either cell type alone.

## Discussion

The above studies establish a therapeutic benefit associated with employing the same CAR T cell to concurrently attack both cancer cells and CAFs. Not only was an immunologically hot tumor more efficiently destroyed in the presence of both bispecific adapters, but an immunologically cold tumor was also successfully suppressed under conditions where neither adapter alone achieved a response. Because tasking the universal CAR T cell with two concurrent killing assignments did not detract from its ability to perform either task independently, we conclude that the CAR T cell still had untapped capacity that could be successful redirected to another cell type. Whether administration of still other bispecific adapters for elimination of other unwanted cells (e.g regulatory T cells, myeloid derived suppressor cells, tumor-associated macrophages, etc.) could have further enhanced therapeutic outcomes was not explored, but this possibility should not be considered unreasonable, since ~100,000 anti-fluorescein CARs are expressed per CAR T cell and only a small number of bridges between a CAR T cell and its target cell are required to induce killing (56). While production of multiple classical CAR T cells dedicated to different tumor cell types could have conceivably achieved the same objectives, their production would have required additional resources, and their activation and proliferation would have only been stimulated by independent engagement of each CAR T cell’s specific target (i.e. not by either CAFs and cancer cells as seen in **Figure 3F**). Thus, use of a universal CAR T cell in combination with multiple adapters can be argued to offer a safe, cost-effective and scalable alternative to the production of multiple CAR T cells. Moreover, if administration of still additional bispecific adapters would allow elimination of still other unwanted stromal cells or perhaps even cancer cells that have downregulated their targeted antigens (52), even more advantages associated with use of a universal CAR T cell would accrue. A potential obstacle to translation of the above CAR T cell technology into humans, however, should also be mentioned. The CAR T cells employed here were first prepared using human T cells and then tested in NSG immunocompromised mice. Although all CAR T cell therapies approved for use in humans to date have followed a similar development path, it will still be important to validate the above results in immune competent animals to assure that immunogenicity issues do arise when universal CAR T cell strategy is translated into humans.

Although multiple distinct subtypes of fibroblasts have been described in the literature, only the myofibroblast subtype has been shown to express FAP (57, 58). Because induction of this FAP is primarily stimulated by TGFβ, which is in turn released in response to tissue damage or stress (e.g. within a wound or tumor), FAP-expressing fibroblasts are primarily observed in traumatized and malignant tissues, where they are thought to stimulate proliferation and regeneration (19–22). Since their contributions to tissue growth may be important to wound healing, the question naturally arises whether a patient recovering from tissue trauma might be retarded in this recovery by treatment with a FAP-targeted CAR T cell therapy. While such risks have not yet been investigated, we suspect that CAR T cell treatment of a cancer may in some cases have to be delayed to allow adequate healing of the concomitant wound.

While the universal CAR T cell approach may come with some advantages, it may also introduce complexities into the clinical application of the therapies. Thus, as noted in **Figure 2**, the concentration dependence of bispecific adapter-mediated killing follows a bell-shaped curve, where target cell engagement/killing first increases and then decreases with increasing adapter concentration. While the plateau of maximum potency is generally seen to extend over an adapter concentration range of two orders of magnitude, the ideal concentration may still not be the same for all patients. And although this complexity may at first appear undesirable, closer scrutiny should reveal that it could also become advantageous, since it would permit sensitive control of the therapy for each patient. Thus, simply lowering the bispecific adapter concentration has been shown to prevent a cytokine storm (50), and decreasing adapter dosing frequency has been similarly documented to avoid CAR T cell exhaustion (10–12, 50). When considered together with the user’s ability to independently adjust the composition of adapters to address each tumor’s cancer and stromal cell composition, these complexities could become tools for personalization. Thus, with the expanding data sets expected to emerge from treatment of increasingly more patients, artificial intelligence methods should enable an informed selection of an optimal adapter composition, concentration, and dosing frequency for each patient, thereby allowing each patient to be treated with a highly personalized therapy.

## Methods

### Cell culture

Hs894 cells (Cat# CRL-7631_FL), 3T3 cells (Cat# CRL-1658), and MDA-MB-231 cells (Cat# HTB-26), KB cells (Cat# CRL-3596) were purchased from ATCC. Hs894 and 3T3 cells were cultured in DMEM medium (Gibco, Cat# 11995073), while MDA-MB-231 and KB cells were cultured in folate-free RPMI 1640 medium (Gibco, Cat# 27016021), all containing 10% heat-inactivated fetal bovine serum and 1% penicillin-streptomycin in 5% CO_2_ at 37°C.

### Generation of human anti-fluorescein CAR T cells

Human peripheral blood mononuclear cells (PBMCs) were isolated from fresh peripheral blood samples obtained from healthy donors using Ficoll (GE Healthcare Lifesciences) density gradient centrifugation. CD3^+^ T cells were then isolated with an EasySep Human T-Cell Isolation Kit (STEMCELL Technologies) and cultured in TexMACS medium (Miltenyi Biotec) supplemented with 1% penicillin-streptomycin, 5% human serum (Valley Biomedical), and human IL-2 (100 IU/mL, Miltenyi Biotec). T cells were count and maintained at 0.5 × 10^6 cells/mL. Human anti-fluorescein CAR T cells were generated using a lentiviral vector protocol as previously described (12, 50–52).

### Animal husbandry

Six- to eight-week-old female NSG mice were purchased from Jackson Laboratory (Stain# 005557) and used for all live animal studies. Mice were housed in accordance with protocols approved by Purdue University Animal Care and Use Committee. Water and folate-deficient chow (Envigo, Cat#TD.00434) were freely available.

### Analysis of CAFs and human CAR T cells in solid tumors

Mice were subcutaneously injected with MDA-MB-231 + Hs894 cell mixtures (3 million cells each/mouse), or KB cells (1 million cells/mouse), and tumor sizes were measured every other day. Mice were sacrificed when control tumors reached ~1500mm^3^ for MDA-MB-231 + Hs894 tumors, and 900 mm^3^ for KB tumors. Part of each tumor was then dissociated with tumor dissociation kit (Miltenyi, Cat# 130-095-929) and erythrocytes were depleted using RBC lysis buffer. After two washes with cold PBS, the resulting single cell suspensions were analyzed by flow cytometry for the desired phenotypic markers (**Supplementary Table S1**). The remaining portion of each tumor was fixed in 4% formalin, followed by storage in 70% ethanol. Subsequent paraffin and immunohistochemistry staining was performed by the Purdue Histology Research Laboratory.

### Analysis of human CAR T cell abundances in mouse peripheral blood

For analysis of CAR T cells from the peripheral blood of treated mice, peripheral blood was collected by cardiac puncture prior to washing 3x in PBS. Erythrocytes in the resulting cell suspensions were then lysed using RBC lysis buffer and residual cells were washed 2x in PBS. The washed PBMCs were then stained with the desired antibodies and analyzed by flow cytometry (**Supplementary Table S1**) for quantitation of CAR T cell numbers in the blood.

### Flow cytometry

Single cell suspensions obtained as described from murine blood or tumor samples were first stained with Zombie Violet (BioLegend, Cat#423114) and then washed 2x with PBS prior to incubation with anti-mouse TruStain FcX™ (BioLegend, Cat#101319) and anti-human TruStain FcX™ (BioLegend, Cat# 422301), respectively, to block nonspecific Fc domain binding. Cells were then stained with the desired antibodies listed in **Supplementary Table S1** and washed 2x with PBS. After washing, cells were resuspended in FACS buffer and examined using an Attune NxT flow cytometer prior to analysis of the data using Attune Cytometric Software and FlowJo V10.

### ELISA analysis of cytokines from mice studies and *in vitro* killing assays

Human IFNγ was quantified using an ELISA MAX™ Deluxe Set (BioLegend, Cat# 430116) kit following manufacturer’s protocol.

### Analysis of anti-FL CAR T cell killing of different cell types

Hs894, 3T3-mFAP, MDA-MB-231-hFAP and parental MDA-MB-231 cells were stained with viability dye (Sartorius, #4839) and seeded onto 96 wells plates (~8000 cells/well for MDA-MB-231 cells and 3T3-mFAP cells, ~5000 cells/well for Hs894 cells), after which anti-FL CAR T cells were then added at a 1:3 effector to target cell (E:T) ratio for MDA-MB-231 cells, and 1:2 E:T ratio for both Hs894 and 3T3-mFAP fibroblasts. To initiate CAR T cell killing, increasing concentrations (0 nM-1000nM) of FAP8-FL bispecific adaptor were added and the incubation was continued for 48 hours. Target cell lysis was then quantitated by dislodging the surviving cells with 0.25% trypsin, washing them twice in complete cell culture media, and counting surviving cells by flow cytometry. Percent target cell lysis was determined by comparing treatment wells to control wells with target cells alone (18, 59).

### Binding of bispecific adapters to anti-FL CAR and tumor antigens on cancer cells

To evaluate the binding affinity of folate-FL and FAP8-FL for the anti-FL scFv on the CAR T cell and its tumor antigen on the cancer cell, folate-FL and FAP8-FL were dissolved in PBS. We quantitated folate-FL binding to the CAR T cells by analyzing displacement of fluorescein-AlexaFluor647 (FL-AF647) from the anti-FL CAR T cells by FAP8-FL. For this purpose, CAR T cells were incubated with various concentrations of FL-AF647 for 1 hour at room temperature, followed by washing 3 times with PBS + 2% FBS and measurement of cell-associated AF647 mean fluorescence intensity by flow cytometry (52). To confirm the specificity of bispecific adapter binding to anti-FL CAR T cells, CAR T cells were also incubated with FL-AF647 in the presence of 100x excess competitive ligands for 1 hour at room temperature. For analysis of the binding affinity of folate-FL and FAP8-FL for its tumor antigen on the cancer cells, increasing concentrations of each of bispecific adapters were incubated with either KB cells (for folate-FL) or MDA-MB-231-hFAP cells (for FAP8-FL) for 1 hour at room temperature. After incubation, cancer cells were washed 3x with PBS + 2% FBS and analyzed by flow cytometry (50, 52). The GraphPad Prism version 10 software was used to analyze binding affinity.

### Binding of FAP8-FL by confocal microscopy

To qualitatively determine the binding of FAP8-FL to human FAP and murine FAP qualitatively under confocal microscopy, MDA-MB-231-hFAP, MDA-MB-231-mFAP, and parental MDA-MB-231 cells were pre-seeded in confocal chambers (Thermo Scientific, Cat# 155360) using complete RPMI at a density of 0.1 million cells per well. The cells were incubated with 100 nM of FAP8-FL at room temperature for 1 hour, followed by three washes with PBS + 2% FBS, and then processed for confocal microscopy measuring fluorescein signals.

## Data Availability

The original contributions presented in the study are included in the article/**Supplementary Material**. Further inquiries can be directed to the corresponding author.
