# Supplementary material for: Use of a universal targeting CAR T cell to simultaneously kill cancer cells and cancer-associated fibroblasts
Source: Front Immunol. 2025 Feb 17;16:1539265. doi: 10.3389/fimmu.2025.1539265 (PMC11873807; doi:10.3389/fimmu.2025.1539265)
Supplement: Supplementary file 1 [file DataSheet1.docx]

**Supplemental information:**

**Table S1: Staining markers and antibody list for flow cytometry**

| Corresponding figure | Cell type / markers | Antibody |
| --- | --- | --- |
| Figure 3E | Hs894 fibroblasts:  Zombie violet-negative, CD45-negative, human FAP-positive | anti-mouse CD45-PE (Biolegend, Cat# 103106),  anti-human FAP-APC (R&D system, Cat# FAB3715A-100) |
| Figure 3F & 3G, 5E & 5F | Human CAR T cells:  Zombie violet-negative, human CD3-positive, anti-CAR monoclonal antibody positive | anti-human CD3-PE/Cy5 (Biolegend, Cat# 300410),  anti-CAR-APC (Provided by Umoja Biopharma) |
| Figure S4 | Staining for human FAP and mouse FAP expressing cells | Anti-human FAP-APC (R&D system, Cat# FAB3715A-100),  Anti-mouse FAP (R&D system, Cat#MAB9727-100) + APC conjugated secondary antibody (R&D system, Cat# F0113) |

**
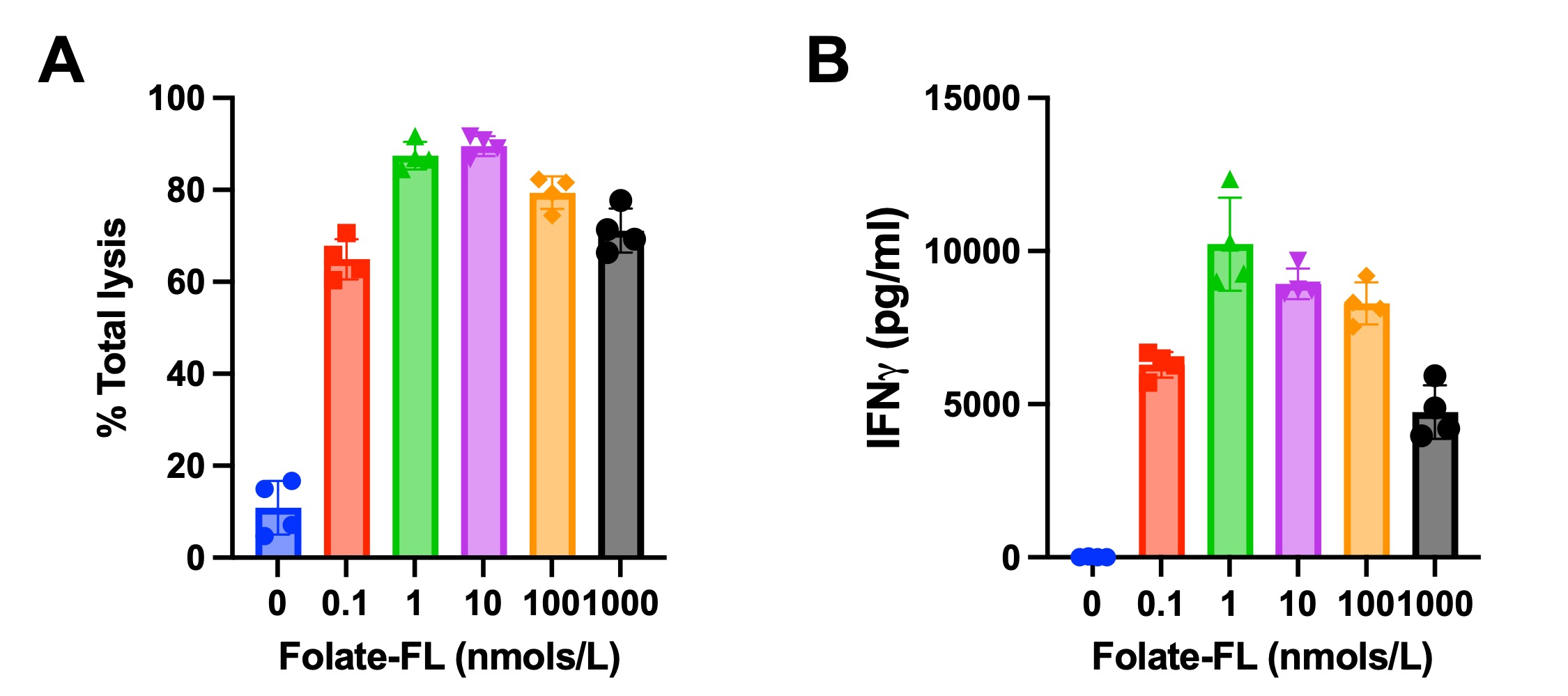
**

**Figure S1. *In vitro* killing of folate receptor over-expressing KB cells.** Anti-FL CAR T cells were added to KB cells at a E:T ratio of 1:3, and folate-FL was added at the different concentration indicated. Cells were then co-cultured for 48hrs before analysis as described in Methods section for (**A**) total killing of KB cells by anti-FL CAR T cells, and (**B**) IFN-gamma cytokine release due to CAR T cell activation. Coculturing anti-FL CAR T cells with KB cells in the absence of folate-FL (0 nmol/L) was used as a negative control. More detailed information on this study can be found in our previous publications. (50, 51). Mean ± SD, n=3.

**
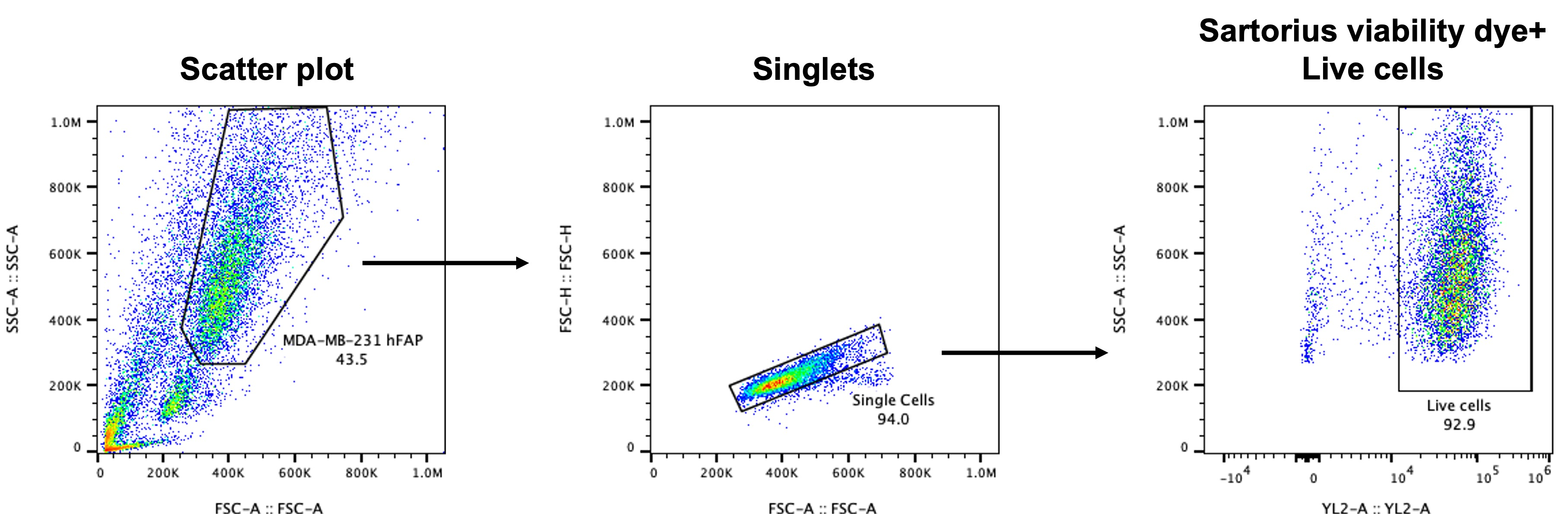
**

**Figure S2. Flow cytometry gating of live cells from *in vitro* killing assay.** Cells were collected from coculture killing assay as described in Methods section and analyzed by flow cytometry. Live cells were determined by Sartorius viability dye positive cells.


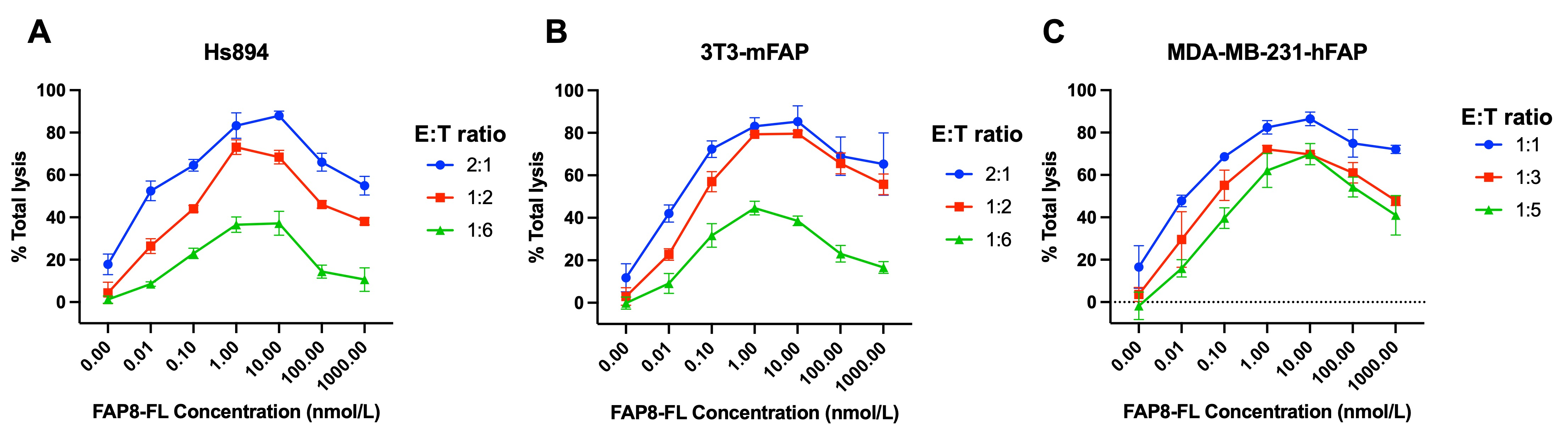


**Figure S3. Effector to target cell titration for *in vitro* killing assay on FAP expressing cells.** Killing of (**A**) Hs894, (**B**) 3T3-mFAP, and (**C**) MDA-MB-231-hFAP under different effector to target (E:T) ratio was performed (n=3) as described in Methods section. Results of representative effector to target ratio was selected and presented in Figure 2. Mean ± SD, n=3.


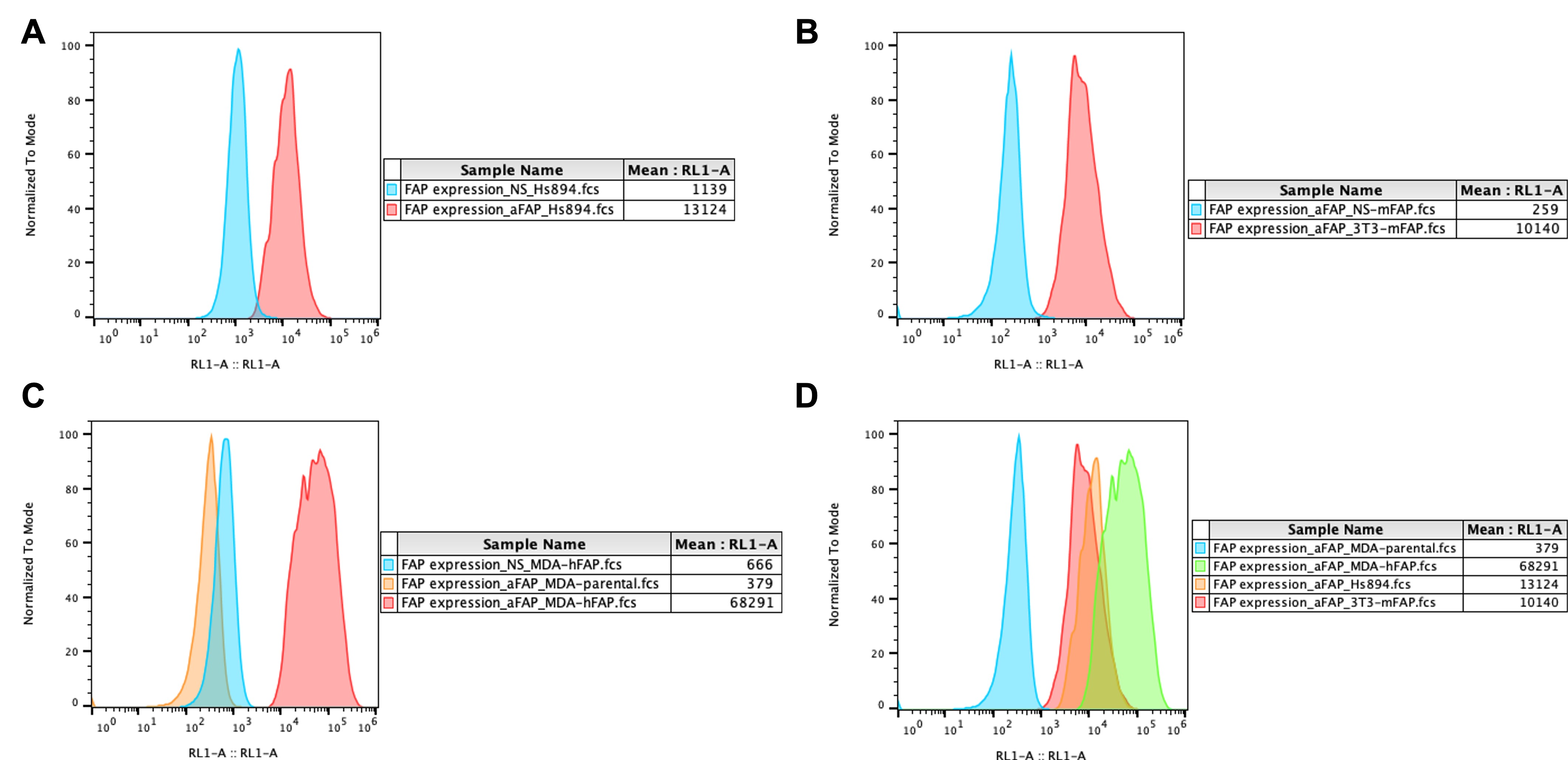


**Figure S4. FAP expression level of FAP-expressing cell lines used in in vitro killing assay.** FAP-expressing cells shown above were stained by either anti-human FAP antibody or anti-mouse FAP antibody (shown in table S1) on ice for 30mins, washed 3 times with PBS + 2% FBS, then proceed to flow cytometry for detection of FAP expression. FAP expression of (**A**) Hs894 CAFs, (**B**) 3T3-mFAP mouse fibroblasts, (**C**) MDA-MB-231-hFAP cells were determined with non staining controls. **Panel D** shows the overlay of FAP expressing cells using parental MDA-MB-231 cells with no FAP expression as control.

**
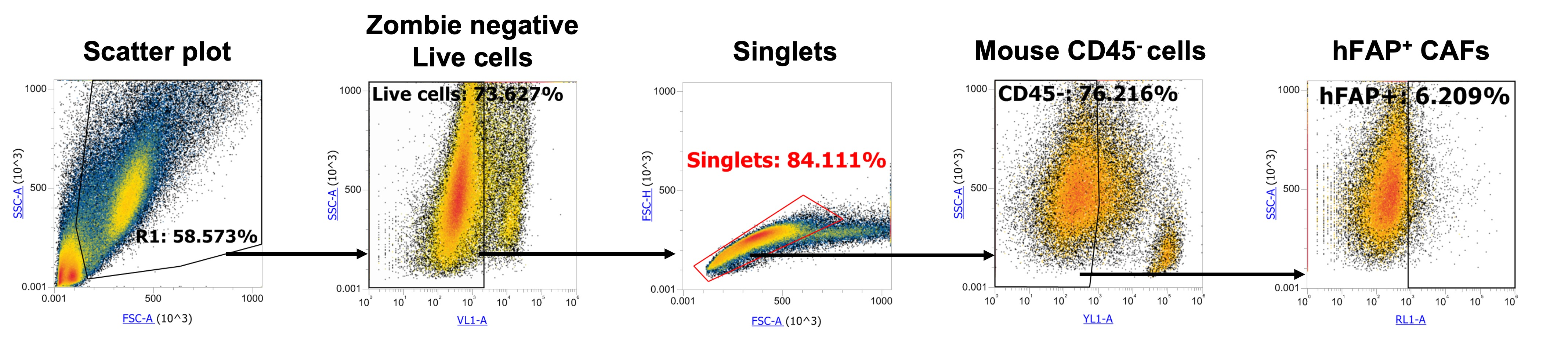
Figure S5.** **Analysis of Hs894 CAF abundances in MDA-MB-231+Hs894 tumors.** MDA-MB-231+Hs894 were digested as described in Methods section. Cells were then stained with a live/dead cell Zombie violet stain and treated with Fc receptor blockers prior to staining for anti-mouse CD45 negative and human FAP positive for Hs894 human CAFs cells.


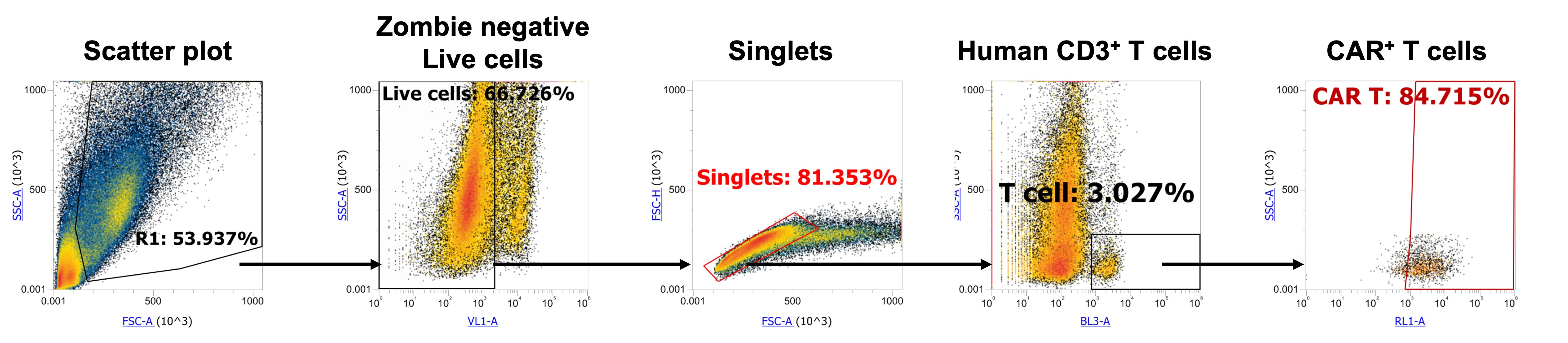
**Figure S6.** **Analysis of human CAR T cell abundances in KB and MDA-MB-231+Hs894 tumors.** MDA-MB-231+Hs894 or KB were digested as described in Methods section. Cells were then stained with a live/dead cell Zombie violet stain and treated with Fc receptor blockers prior to staining with anti-human CD3 antibody for human T cells and a monoclonal antibody that recognizes the anti-fluorescein CAR for CAR T cells.

**
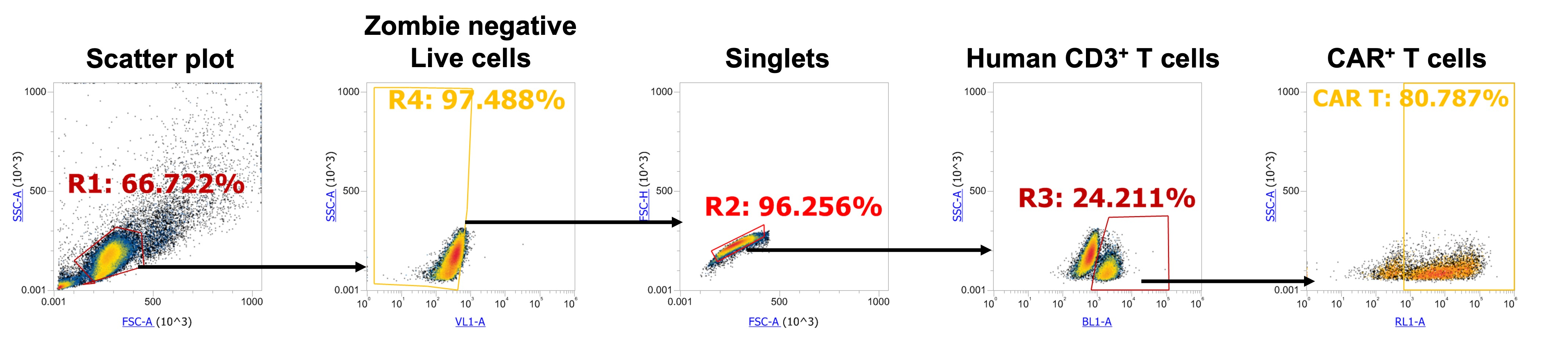
Figure S7.** **Analysis of human CAR T cells in mouse peripheral blood.** Mouse pheripheral blood was collected as described in Methods section. Washed PBMCs were then stained with live/dead cell marker Zombie violet and Fc receptor blockers prior to staining with human CD3 antibody for human T cells + monoclonal antibody for anti-fluorescein CAR for anti-fluorescein CAR T cells.


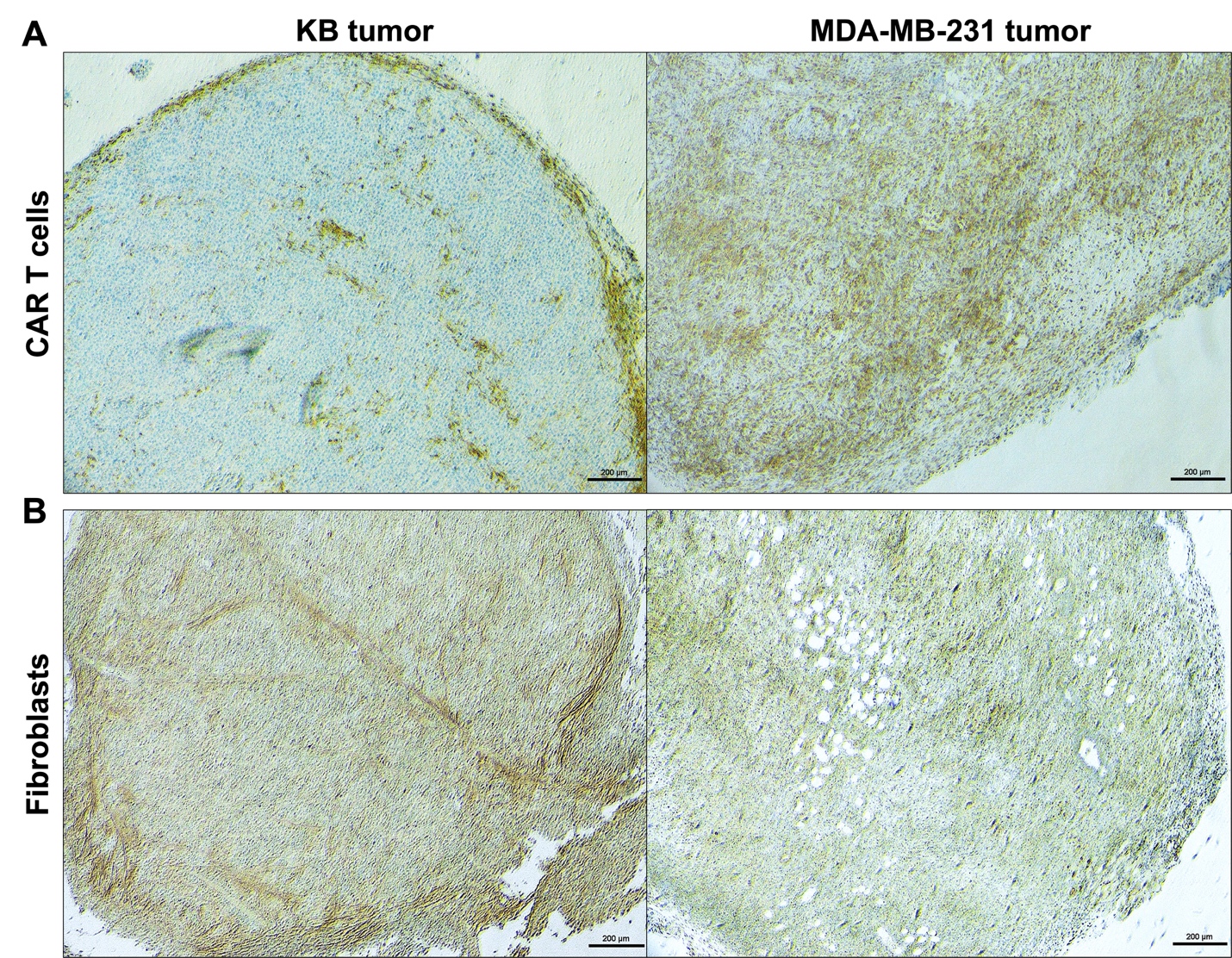


**Figure S8.** (**A**) IHC staining of immunological cold KB tumor and hot MDA-MB-231 tumor with anti-human CD3 antibody (staining for human CAR T cells) and (**B**) with anti-mouse FAP antibody (staining for FAP^+^CAFs).
